# Editorial: Neurotological consequences of long COVID

**DOI:** 10.3389/fneur.2022.1087896

**Published:** 2022-11-21

**Authors:** Haúla Faruk Haider, Agnieszka J. Szczepek

**Affiliations:** ^1^ENT Department Nova Medical School, Hospital Cuf Infante Santo, Lisbon, Portugal; ^2^Department of Otorhinolaryngology, Head and Neck Surgery, Charité-Universitätsmedizin Berlin, Corporate Member of Freie Universität Berlin, Berlin Institute of Health, Humboldt-Universität zu Berlin, Berlin, Germany; ^3^Faculty of Medicine and Health Sciences, University of Zielona Góra, Zielona Góra, Poland

**Keywords:** COVID-19, post-COVID sequelae, neurotology, hearing loss, balance disorders, long COVID

COVID-19 took the world by surprise at the beginning of 2020. Until today, serious flu symptoms, severe pneumonia, lung or kidney failure, and death caused by the SARS-CoV-2 virus intimidate populations worldwide. After a few months of the pandemic, it became clear that not all those who underwent COVID-19 fully recovered and that the long-term affected organs must not necessarily belong to the respiratory tract. It was when the term “long-COVID” or “post-COVID” was coined. There have been many attempts to classify and systematize post-COVID symptoms. The literature reports on that topic have been analyzed by Lopez-Leon et al. in a systematic review and meta-analysis (1). That analysis demonstrated more than 50 symptoms associated with COVID-19 sequelae. Importantly, Lopez-Leon et al. (1) found that roughly 80% of persons who contracted COVID-19 reported having one or more symptoms for more than two and up to 16 weeks after recovery. One of these symptoms turned out to be hearing loss and/or tinnitus, reported by 15% of persons as a long-term consequence of COVID-19. Another continuing symptom affecting 3% of post-COVID-19 patients was dizziness.

The Research Topic “*Neurotological consequences of long COVID*” at Frontiers in Neuro-Otology was created as an exchange platform for clinicians and scientists specializing in otology, audiology, and neurology and interested in the topic of long COVID affecting the inner ear. Four research groups have addressed the occurrence and characteristics of tinnitus, vertigo, and dizziness in the context of COVID-19 (Figure 1).

**Figure 1:**
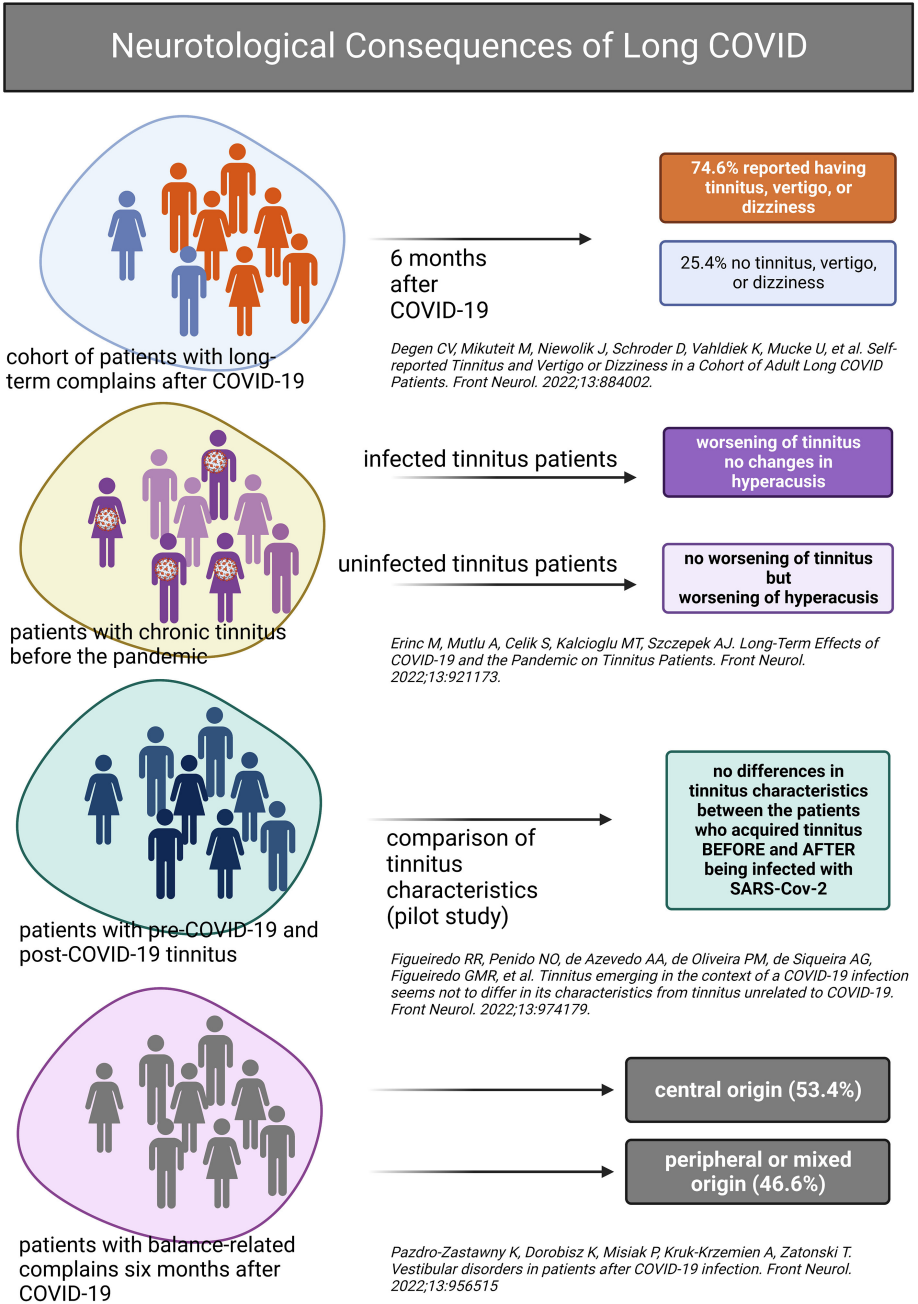
Graphical summary of the main findings presented in the Research Topic “*Neurotological consequences of long COVID*.” Created with BioRender.com.

Degen et al. in their work “*Self-reported tinnitus and vertigo or dizziness in a cohort of adult long COVID patients*” presented data collected between September 2021 and January 2022 from a cohort of 1,082 patients (15% of them fully vaccinated) complaining of symptoms on average 43.2 weeks after recovery from SARS-CoV-2 infection. Sixty percent of long COVID patients complained of vertigo or dizziness and, on average, rated their discomfort on a scale from 0 (no discomfort) to 10 (greatest discomfort) as being 4.6 (SD 2.7). In the same cohort, 30.6% reported having tinnitus with discomfort

rated on average 4.8 (SD 3.0) on a scale from 0 to 10. Twenty-four percent reported having all symptoms, and 25.4% had no tinnitus, vertigo, or dizziness. The results of Degen et al. differ from those of Lopez-Leon et al. (1) in that Degen et al. observed a twofold tinnitus prevalence among individuals with long COVID, as compared to the meta-analysis. However, the meta-analysis was submitted for publication in March 2021. In contrast, the cohort study of long COVID patients was submitted in February 2022 and based on data collection initiated in September 2021 (is still ongoing). Within 1 year, not only has SARS-CoV-2 changed but also society's awareness of long COVID. In addition, Degen's study mainly focused on neurotological symptoms associated with long COVID. In contrast, Lopez-Leon collected studies of various designs, sometimes focusing on all health aspects affected by long COVID and sometimes on specific, often non-neurotological issues.

In the second (pilot) study, published in our Research Topic, “*Tinnitus emerging in the context of a COVID-19 infection seems not to differ in its characteristics from tinnitus unrelated to COVID-19*,” patients of the ENT clinic who contracted COVID-19 (as per RT-PCR) were recruited between November 2020 and November 2021 and split into patients without (*n* = 33) and with tinnitus (*n* = 24). The latter group was subdivided into patients with chronic tinnitus before the SARS-CoV-2 infection (*n* = 13) and those who reported tinnitus after the SARS-CoV-2 infection (*n* = 11). The three groups were compared regarding tinnitus severity, hearing abilities, COVID-19 symptoms, and treatment. Figueiredo et al. found no difference in tinnitus characteristics or hearing abilities between the two tinnitus groups and concluded that tinnitus with the post-COVID-19 onset and pre-COVID-19 onset do not differ. This exciting pilot study would be worth extending regarding the sample size and observation period.

The main question of the third study, “*Long-term effects of COVID-19 and the pandemic on tinnitus patients*,” was *if* and *how* the patients diagnosed with chronic tinnitus before the pandemic reacted to SARS-CoV-2 infection or/and to the situation created by the pandemic in terms of tinnitus-relevant parameters. Erinc et al. collected data from 96 patients with chronic tinnitus in January 2022 and compared it to the pre-pandemic values, splitting the tinnitus sample into COVID-19 positive and negative. Their main finding was that the tinnitus-related parameters (Tinnitus Handicap Inventory THI and visual analog scales) worsen in patients with chronic tinnitus after infection with SARS-CoV-2. In contrast, patients who did not contract COVID-19 reported worsening of their hyperacusis, as per Hyperacusis Questionnaire HQ.

Last manuscript belonging to our Research Topic, “*Vestibular disorders in patients after COVID-19 infection*,” deals with vestibular consequences of COVID-19. In that work, a group of patients who reported vertigo up to 6 months after contracting COVID-19 was studied (Pazdro-Zastawny et al.). Vertigo of central origin was diagnosed in more than half of the patients, whereas the remaining subjects had peripheral or mixed vestibular symptoms, implicating vertigo as a potential long-term sequel of COVID-19.

As we write this editorial, the pandemic has been going on for almost 3 years, and although vaccines are effective in priming the immune system to fight the virus (2), and sophisticated modeling helps predict the subsequent waves of infection (3), but there is no forecast that this global problem will end soon. Neurotological symptoms are neither leading during primary SARS-CoV-2 infection nor among long-term post-COVID symptoms. Nevertheless, hearing loss, tinnitus, hyperacusis, or vertigo significantly reduce the quality of life and negatively affect interpersonal communication and social life. All these conditions were documented in our RT as the long-term consequences of COVID-19, but many questions remain open. What factors predispose to the development of post-COVID neurotological symptoms? Do vaccines protect against the neurotologic COVID sequel? Is there a link between the virus strain and the neurotological consequences of COVID-19? To answer these and many other questions, we encourage the community to continue to collect and analyze clinical data and conduct translational research that may provide some solutions in the future.

## Author contributions

HH: concept, writing first draft, and editing. AS: concept, writing first draft, editing, and visualization. Both authors contributed to the article and approved the submitted version.

## Conflict of interest

The authors declare that the research was conducted in the absence of any commercial or financial relationships that could be construed as a potential conflict of interest.

## Publisher's note

All claims expressed in this article are solely those of the authors and do not necessarily represent those of their affiliated organizations, or those of the publisher, the editors and the reviewers. Any product that may be evaluated in this article, or claim that may be made by its manufacturer, is not guaranteed or endorsed by the publisher.
